# Peptide selection via phage display to inhibit *Leishmania*-macrophage interactions

**DOI:** 10.3389/fmicb.2024.1362252

**Published:** 2024-02-27

**Authors:** Juliane Buzzon Meneghesso Verga, Márcia A. S. Graminha, Marcelo Jacobs-Lorena, Sung-Jae Cha

**Affiliations:** ^1^Department of Clinical Analysis, School of Pharmaceutical Sciences, São Paulo State University (UNESP), Araraquara, Brazil; ^2^Molecular Microbiology & Immunology, Johns Hopkins Malaria Research Institute, Johns Hopkins School of Public Health, Baltimore, MD, United States; ^3^Department of Medical Sciences, Mercer University School of Medicine, Macon, GA, United States

**Keywords:** phage display 12-mer peptide library, Leishmania-macrophage interaction, ligand-receptor interaction, metacyclic promastigote, visceral leishmaniasis

## Abstract

**Introduction:**

Leishmaniasis comprises a complex group of diseases caused by protozoan parasites from the *Leishmania* genus, presenting a significant threat to human health. Infection starts by the release into the skin of metacyclic promastigote (MP) form of the parasite by an infected sand fly. Soon after their release, the MPs enter a phagocytic host cell. This study focuses on finding peptides that can inhibit MP-phagocytic host cell interaction.

**Methods:**

We used a phage display library to screen for peptides that bind to the surface of *L. amazonensis* (causative agent for cutaneous leishmaniasis) and *L. infantum* (causative agent for cutaneous and visceral leishmaniasis) MPs. Candidate peptide binding to the MP surface and inhibition of parasite-host cell interaction were tested *in vitro*. Peptide Inhibition of visceral leishmaniasis development was assessed in BALB/c mice.

**Results:**

The selected L. amazonensis binding peptide (La1) and the *L. infantum* binding peptide (Li1) inhibited 44% of parasite internalization into THP-1 macrophage-like cells *in vitro*. While inhibition of internalization by La1 was specific to *L. amazonensis*, Li1 was effective in inhibiting internalization of both parasite species. Importantly, Li1 inhibited *L. infantum* spleen and liver infection of BALB/c mice by 84%.

**Conclusion:**

We identified one peptide that specifically inhibits *L. amazonensis* MP infection of host cells and another that inhibits both, *L. amazonensis* and *L. infantum*, MP infection. Our findings suggest a promising path for the development of new treatments and prevention of leishmaniasis.

## Introduction

Leishmaniasis is a neglected tropical infectious disease caused by *Leishmania*, a protozoan parasite transmitted by phlebotomine sand flies (World Health Organization [WHO], 2023). Leishmaniasis affects ∼350 million people in 102 endemic countries, accounting for 1.3 million annual cases and 20,000 to 30,000 deaths every year (Pan American Health Organization [PAHO], 2019). Over 20 *Leishmania* species can infect humans, manifesting through three main forms: cutaneous, mucocutaneous, and visceral leishmaniasis (Pan American Health Organization [PAHO], 2019; Mann et al., 2021; World Health Organization [WHO], 2023). Out of these, visceral leishmaniasis (VL) is the most devastating form, accounting for ∼100,000 cases per year (Matlashewski et al., 2013; Pan American Health Organization [PAHO], 2019; CDC, 2020; Mann et al., 2021). Cutaneous leishmaniasis (CL) is the most common form and causes ulcer-like skin lesions on exposed parts of the body, leaving life-long scars, serious disability, and stigma. Currently, there are an estimated 700,000 to 1.2 million annual CL cases; the USA has been classified as endemic for leishmaniasis since 2015 due to autochthonous CL cases (McIlwee et al., 2018). Mucocutaneous leishmaniasis (ML) is caused by infection in the nasal and oral cavity, giving rise to strong inflammation and facial disfiguration (Pan American Health Organization [PAHO], 2019; Mann et al., 2021; World Health Organization [WHO], 2023). Only few treatments for leishmaniasis exist and can be toxic (Pan American Health Organization [PAHO], 2019; Mann et al., 2021; World Health Organization [WHO], 2023). Thus, the development of novel drugs and vaccines for leishmaniasis control is high priority. The treatment of leishmaniasis lacks a universal approach. Traditionally, antimonials administered through injections have been the primary therapy, but their use is associated with severe side effects. Liposomal amphotericin B is effective for certain forms of leishmaniasis but requires skilled administration. Miltefosine is the only available oral drug. Treatment guidelines vary across regions and depend on factors such as leishmaniasis type, causative parasite, patient immune status, and local therapeutic availability (eBioMedicine., 2023).

Vertebrate infection by *Leishmania* parasites is initiated by the delivery of flagellated motile MPs from the anterior gut of the infected sand fly to the bite site (Serafim et al., 2021). MPs enter phagocytic mononuclear cells at the bite site, such as neutrophils, monocytes, dendritic cells, or skin macrophages (Ueno and Wilson, 2012; Carneiro and Peters, 2021). Within the phagolysosome, MPs lose motility as their flagella retract during transforming into amastigotes, and then multiply and develop within the reticuloendothelial system. Amastigotes can travel through the circulatory or lymphatic system to cause mucosal or visceral disease (Ueno and Wilson, 2012; Carneiro and Peters, 2021). Therefore, MP infection of phagocytic host cells is the first obligatory step for disease development and a prime target for protection via immunization. Repeated probing by the female sand fly at the bite site causes laceration of capillary vessels and the formation of a blood pool which serves as the initial site of MP entry into host phagocytic cells. Tissue injury and sand fly derived factors (such as saliva, microbiome, and promastigote secretory gel) activate the release of chemokines that activate infiltration of host innate immune cells, such as neutrophils and monocytes (Serafim et al., 2021). Of note, MP infection of phagocytic host cells is the first obligatory step for disease development and a prime target for protection via immunization.

The final destination of parasites from each species is different, causing parasite species-specific disease forms. For instance, *L. infantum* develops VL, occasionally causing CL, but it is not associated with ML; *L. amazonensis* develops CL or ML, rarely VL (Carneiro and Peters, 2021; Mann et al., 2021). Although it is controversial whether MP entry into host cells is either passive or active (Rodríguez et al., 2006, 2011; Mayor and Pagano, 2007; Forestier et al., 2011; Walker et al., 2014; Andrade, 2019; Cavalcante-Costa et al., 2019; Valigurová and Kolářová, 2023), MP entry into phagocytic host cells is mediated by receptor-mediated phagocytosis (Carneiro and Peters, 2021). Other host cells and different *Leishmania* species display various surface molecules, leading to unique parasite-host receptor interactions for each [parasite species–host cell] pair (Ueno and Wilson, 2012). Moreover, MPs of a given species can interact with different receptors from different host cells, eliciting other downstream host cell functions (Ueno and Wilson, 2012; Carneiro and Peters, 2021). Therefore, characterization of the initial ligand-receptor interaction of each parasite species is likely to lead to mechanistic insights into downstream disease pathways (Carneiro and Peters, 2021; Mann et al., 2021). Molecular identification of ligand-receptor interaction for parasite-host cell recognition can be initiated by selecting peptides that interfere with this specific interaction (Ghosh et al., 2009, 2011; Cha et al., 2015, 2016, 2021; de Paula et al., 2022). Here, we used a phage peptide display approach to select peptides that bind to the surface of *L. amazonensis*, *L. infantum*, or both MP species, the parasite form responsible for initiating mammalian host infection. The selected peptides inhibited *in vitro* host cell internalization and *L. infantum* infection *in vivo*.

## Materials and methods

### *Leishmania* and THP-1 cell culture

*Leishmania infantum* (MHOM/MA/67/ITMAP-263) and *L. amazonensis* (MPRO/BR/1972/M1841-LV-79), parasites were cultivated at 26°C in Schneider’s Drosophila Medium (Sigma S0146- with glutamine) supplemented with 10% FBS, 5% penicillin (100 U/mL)–streptomycin (100 μg/mL), and hemin (5 mg/mL) only for *L. infantum*.

THP-1 human monocyte cells were cultured in RPMI medium supplemented with 10% fetal bovine serum and induced to differentiate into macrophages-like cells by treatment of PMA (Sigma-Aldrich; 1,000-fold dilution of a 0.1 mM stock in DMSO) and incubated for 4 days at 37°C (Auwerx, 1991; Traore et al., 2005; Cha et al., 2015).

### Enrichment of MPs with Ficoll density gradient medium

*Leishmania amazonensis* and *L. infantum* were cultured for 8 days and then MPs were enriched by centrifugation using Ficoll-Paque^®^ PLUS (GE Healthcare) gradient using 0, 10 and 20% solution. This step reduces contaminants, including debris and dead cells, without decreasing parasite viability and infectivity. MPs were quantified by hemocytometer counting following the protocol (Späth and Beverley, 2001). Parasite morphology was evaluated under light microscopy and were considered metacyclic promastigote when the flagellum length represents twice the size of body cell (da Luz et al., 2009; Sunter and Gull, 2017). Despite the purification, small amounts of other morphologies could be included in the parasite pool. However, during our observations MP form was dominant.

### Phage selection

In our study, we employed a filamentous M13 (f88.4) phage library that display 12 amino acid random peptide (Bonnycastle et al., 1996). Initial screening mixed 10^9^ MPs with a total of 10^11^ library phages in 100 μl of culture medium. After 30 min incubation at 26°C, unbound and loosely bound phages were removed by washing with PBS six times. Bound phages were recovered by adding host *Escherichia coli* cells, followed by propagation of the phages in the added bacteria. This selection was repeated three more times, each time with the enriched phage population of the previous round. After the fourth round, the recovered phages were plated, and 32 random colonies were picked for sequencing of the DNA insert as previously described (Bonnycastle et al., 1996).

### Synthetic La1, Li1, Li1scr peptide

La1 (biotin- KCRQWWLDSRCG), Li1 (biotin- ECKRARSAPNCN) and Li1scr (biotin-ACRKNRESNACP, the same amino acid composition as Li1 with a different sequence) peptides were synthesized by Peptide 2.0, Inc.

### Phage/peptide binding assay and *in vitro* inhibition assay for MP-host cell interaction

We used separate titrations for phage/peptide binding assays (IFAs) and for inhibition assays. For phage/peptide binding assays, MPs of each species were fixed in 4% paraformaldehyde in PBS overnight at 4°C. Fixed MPs were blocked with 4% bovine serum albumin in PBS for 1 h to reduce non-specific binding. Subsequently, MPs were incubated either with 2 × 10^6^ phages/μl or with 0.2 mg/ml of a synthetic peptide in PBS overnight at 4°C. After thorough washing steps, bound phages and biotinylated peptides were visualized using an anti-M13 phage antibody plus Alexa 488^®^ -conjugated secondary antibody and Alexa 596^®^ -conjugated streptavidin, respectively.

For *in vitro* inhibition assays, MPs of each species were fluorescently labeled using succinimidyl ester of carboxyfluorescein (CFSE) according to the manufacturer’s instructions (Thermo Fisher Scientific). PMA treated THP-1 cells were prepared in an 8-well Lab-Tek II™ chamber slide (Nunc™) with 200 μl medium per chamber. CFSE labeled MPs were transferred into THP-1 cell culture with candidate phages (2 × 10^6^CFU/μl) or peptide (0.5 mg/ml). After 2 h incubation, the number of bound and internalized MPs were determined after triple washes the THP-1 cells with PBS. Using fluorescent microscopy, we observed that internalized MPs lost flagella to transform into a round amastigote form.

### qRT-PCR assays

Total RNA was isolated from an infected mouse liver or a spleen using TRIzol^®^ reagent (Thermo Fisher Scientific). The first-strand cDNA library was synthesized using Superscript III (Invitrogen) and random hexamers (Invitrogen). Relative parasite burden was determined by real-time PCR using a *Leishmania* specific primer set for LINJ_15_0010 histone H4 (Gene ID: 5067885): forward primer, 5′-GCTGAACCCGTCCGAGGT-3′; reverse primer, 5′-TGAGCCCTTTGCCGAACA-3′. *Leishmania* RNA quantity was normalized by mouse GAPDH expression as previously described (Cha et al., 2016). We used a total of 31 BALB/c mice. All animal experimental procedures were consistent with the recommendations of the Panel on Euthanasia of the American Veterinary Medical Association and were approved by the Mercer University Institutional Animal Care and Use Committee.

### Statistics for data analysis

Two tailed Mann-Whitney *U*-test was used for non-parametric analysis in Figures 3–5.

## Results

### Screening a phage display library for peptides that bind to MPs

**FIGURE 1:**
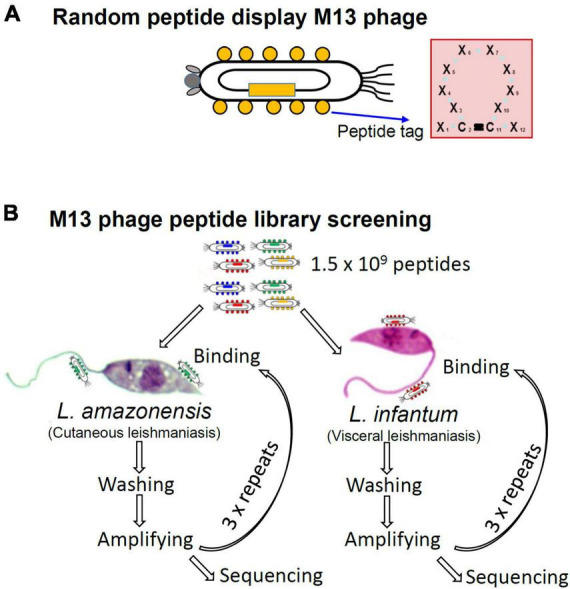
Structure of the phage peptide display library and strategy for screening parasite-binding peptides in search for *Leishmania* MP ligands that inhibit host cell interaction. **(A)** The M13 phage was engineered to display a random 12-amino acid peptides fused to the coat protein pVIII. The second and eleventh amino acids are fixed as cysteines to make a disulfide bond, which makes a circular conformation. **(B)** Library phages, of which complexity is calculated as 1.5 × 10^9^, were incubated with enriched MPs of *L. amazonensis* or *L. infantum* for phages that inhibit MP-host cell interaction. After four repeated panning amino acid sequences of the selected peptide were deduced by DNA sequencing.

Seeking to identify peptides that bind to the surface of *Leishmania* MPs, we employed an M13 phage library (Bonnycastle et al., 1996) that displays random 12-amino acid peptides. The library has an estimated complexity of 1.5 × 10^9^ different peptides. As shown in Figure 1A, library peptides have cysteines at positions two and eleven that form a disulfide bond that gives the peptides a circular conformation. We conducted separate screenings for peptides that bind *L. amazonensis* and *L. infantum* MPs. For initial screenings, 10^9^ MPs of each species were incubated with a total of 10^11^ library phages and phages with low binding affinity were removed by thorough washing. Phages that remained bound to *Leishmania* MPs were recovered by adding host *E. coli* cells for phage amplification. After four rounds of panning, the peptide sequence displayed by the selected phages was determined via DNA sequencing (Figure 1B).

### Properties of MP-binding peptides

**FIGURE 2:**
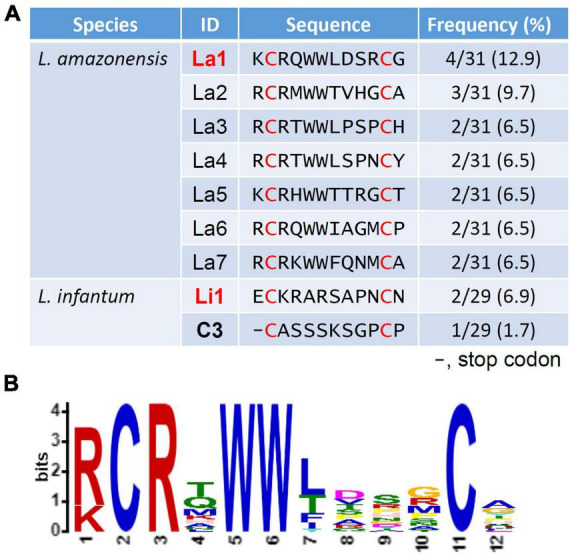
Predicted amino acid sequence of displayed peptides and analysis of the predominant amino acids at each position. **(A)** A total of 32 recombinant phages per species were sequenced after the fourth round of selection. All phages of the library have a cysteine at positions 2 and 11 (red font). One representing peptide of each species (bold red font) was selected for further experiments. Frequency was determined by the number of the selected phages that have an identical insert sequence out of the total number of successful sequences. **(B)** The La1 was selected using MEME analysis (https://meme-suite.org/meme/tools/meme).

Amino acid sequences displayed by phages enriched in the screen of both species are summarized in Figure 2A. A strong peptide enrichment in the *L. amazonensis* screen occurred, where 24 out of 31 sequenced phages displayed peptides with similar traits, having arginine and double tryptophan at the same positions (Figure 2B). One representative recombinant phage from each species was selected for further assays. For L. amazonensis the phage La1 represents 12.9% (4/31) of the total predicted sequences, while for L. infantum the sequence Li1 represents 6.9% (2/29). The C3 phage from the *L. infantum* screen was selected as a negative control, as it has a stop codon at the first amino acid position and is predicted to not display a peptide.

### La1 and Li1 phages bind specifically to MPs and inhibit parasite internalization

**FIGURE 3:**
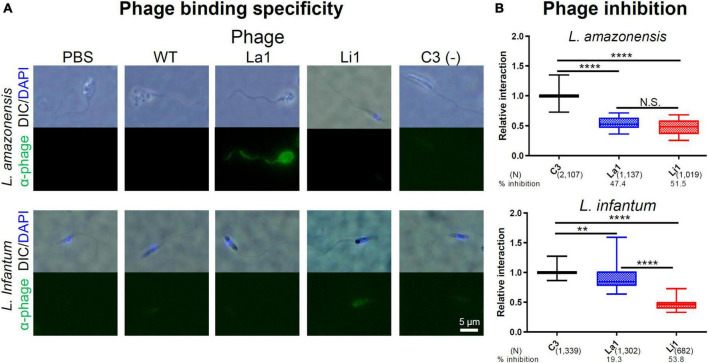
Selected phage binding inhibits parasite-host cell internalization *in vitro*. **(A)** Fixed MPs of each *Leishmania* species, denoted on the left were incubated with 2 × 10^6^ CFU/μl of the selected phages denoted on the top. PBS, C3 phage, and wild type (WT) phage served as negative controls. After washing, bound phages were visualized with an anti-M13 phage antibody and Alexa 488^®^ -conjugated secondary antibodies. **(B)** A total of 1 × 10^5^ CFSE-labeled parasites were added to activated THP-1 macrophage-like cells in an 8-well slide monolayer culture (1 m.o.i.) together with 2 × 10^6^CFU/μL of selected phages denoted on the bottom. The number of attached and internalized parasites, in 100 microscopic fields under 600x magnification, were determined after 2 h at 37°C. Data pooled from five independent experiments. Percent inhibition was determined by comparing to C3 phage-treated group. *P*-values (**P < 0.01; ****P < 0.0001) were determined with the Mann-Whitney *U*-test. Vertical bars: range of the upper and the lower quartile, horizontal lines: medians, (N): number of MPs analyzed.

To determine specificity of phage interaction with the surface of *L. amazonensis* and *L. infantum* MPs, we incubated La1 and Li1 phages with MPs of each species. Figure 3A shows that the La1 phage strongly binds to the *L. amazonensis* MP surface and not to *L. infantum* MPs. The Li1 phage binds to the *L. infantum* cell body only, not to the flagella. No binding above background was detected for the wild type (WT) and C3 phage controls. Specific binding of the selected phages to MPs raised two possible scenarios: (1) the phages bind to a putative MP ligand for host cell entry, or (2) the phages bind to an MP surface molecule unrelated to host cell interaction. In the first scenario, phage binding should result in inhibition of MP host cell entry, since phage occupancy of the ligand would preclude interaction with the presumed host cell receptor. In the second scenario, phage binding should not interfere with MP entry. To distinguish between the two possibilities, the selected phages were incubated for 2 h together with fluorescently labeled MPs and THP-1 human macrophage-like cell cultures. After washing with PBS, bound and internalized MPs were counted. Percent inhibition by the selected phages was determined by comparison with the control C3 phage-treated group. Figure 3B shows that the La1 phage selectively inhibit *L. amazonensis* MP-host cell interaction (47.4% inhibition). Interestingly, the Li1 phage inhibited both species, even though Li1 phage binding to *L. amazonensis* parasites was not detected in the experiments illustrated in Figure 3A. Li1 phages reduced the internalization of *L. amazonensis* MPs by 51.5% and *L. infantum* MPs by 53.8%. These results suggest that the selected phages bind to MP surface molecule(s) required for entry into macrophage cells.

### Synthetic peptides La1 and Li1 inhibit parasite internalization

**FIGURE 4:**
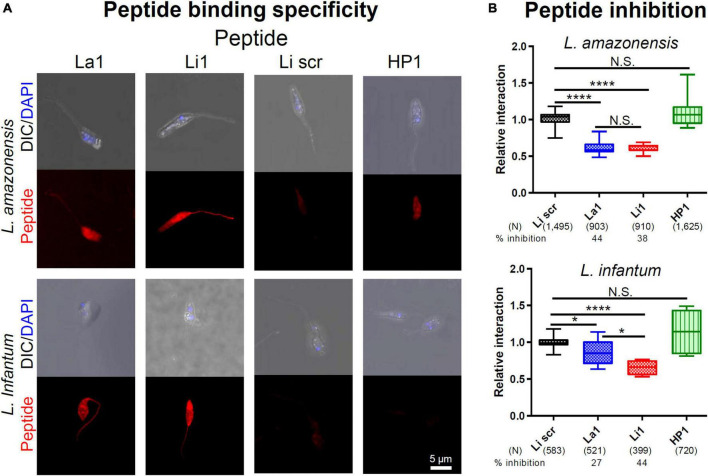
Selected synthetic peptide binding inhibits parasite-host cell interaction *in vitro.* **(A)** Fixed MPs of each *Leishmania* species, denoted on the left, were incubated in PBS with 0.2 mg/ml of the synthetic peptide denoted on the top. Li1scr and the unrelated HP1 peptide served as negative controls. After washing, peptide binding was visualized with an Alexa 596^®^ -conjugated streptavidin. DAPI stained the parasite nuclei. **(B)** A total of 1 × 10^5^ CFSE-labeled parasites were added to activated THP-1 macrophage-like cells in an 8-well slide monolayer culture (1 m.o.i.) together with 0.5 mg/ml of the peptide denoted on the bottom. The number of attached and internalized parasites, in 100 microscopic fields under 600x magnification, were determined after 2 h at 37°C. Data pooled from two independent experiments. Percent inhibition was determined by comparing to Li1scr peptide treated group. *P*-values (*P < 0.05; ****P < 0.0001) were determined with the Mann-Whitney *U*-test. Vertical bars: range of the upper and the lower quartile, horizontal lines: medians, (N): number of MPs analyzed.

We also investigated whether inhibition of MP internalization was due to steric hindrance (phage particles have a diameter of 7 nm and a length of 1 μm) or by actual occupancy of MPs’ ligand(s). These experiments were carried out with La1 and Li1 synthetic peptides and used a scrambled version of the Li1 synthetic peptide (Li1scr, ACRKNRESNACP) and the unrelated HP1 synthetic peptide (Cha et al., 2021) as negative controls (Figure 4). As shown in Figure 4A, both La1 and Li1 peptides bound to both parasite species. The binding of control peptides to MPs was limited to the parasite’s body and was moderate compared to the La1 and Li1 peptide binding. To evaluate the potential inhibitory effects of the peptides, fluorescently labeled MP parasites were incubated with 0.5 mg/ml of each biotinylated synthetic peptide in a THP-1 human macrophage-like cell culture for 2 h. In line with the phage inhibition results, the La1 peptide selectively inhibited the internalization of *L. amazonensis* MPs into host cells by 44%, while the Li1 peptide inhibited the internalization of both species: 38% inhibition for *L. amazonensis* and 44% inhibition for *L. infantum*, respectively. The control HP1 peptide did not inhibit parasite-host cell interaction (Figure 4B). We conclude that steric hindrance does not play a role in inhibition by the recombinant phages and that the peptides are the effectors of inhibition of parasite entry into host macrophages.

### Li1 peptide binding to *L. infantum* MPs inhibits VL development in BALB/c mice

**FIGURE 5:**
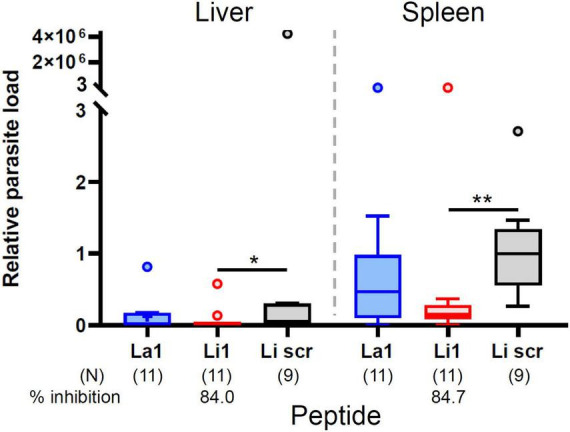
The Li1 peptide inhibits *L. infantum* infection and subsequent visceral leishmaniasis development. Ten million *L. infantum* MP parasites together with 20 μg peptide (denoted in the *X*-axis) in 0.1 ml of PBS were injected into the peritoneum of each BALB/c mouse. One month after injection, mice were sacrificed for quantification of the parasite load in liver and spleen. Parasite load was determined by qRT-PCR. Data pooled from three independent experiments. Each small circle denotes outliers by Tukey’s analysis. Percent inhibition was determined by comparing to the Li1scr peptide treated group. *P*-values (*P < 0.05; **P < 0.01) were determined with the Mann-Whitney *U*-test. Vertical bars: range of the upper and the lower quartile, horizontal lines: medians, (N): number of mice analyzed.

To assess the potential *in vivo* inhibitory effects of the peptides on visceral infection and to corroborate *in vitro* findings, we performed *in vivo* inhibition assays. We incubated 1 × 10^7^ *L. infantum* MPs with 20 μg of each peptide in 100 μl PBS (0.2 mg/ml) for 10 min at room temperature, and this mixture was injected into the peritoneum of BALB/c mice. Infected mice were sacrificed for RNA isolation from liver and spleen to determine parasite burden in each organ 30 days post infection. As shown in Figure 5, the Li1 peptide significantly reduced parasite growth in both organs: 84.7% inhibition in the spleen and 84% inhibition in the liver. La1 peptide has no inhibition of parasite growth in both organs as expected from the *in vitro* inhibition assays in Figure 4B.

## Discussion

We have previously used a phage peptide-display approach using a filamentous M13 (f88.4) phage library to identify receptor-ligand interactions for malaria parasite invasion of the mosquito midgut, of the mosquito salivary glands and of the mammalian liver (Ghosh et al., 2009; Cha et al., 2015, 2016, 2021). Different from the phage libraries that display linear peptides, the f88.4 phage library displays circularized 12-amino acid peptides that maintain stable conformation. In this study, we screened the same phage library for peptides with high affinity to the surface of *L. amazonensis* and *L. infantum* MPs of which binding inhibits MP-host cell interaction. Although main surface protein GP63 has been reported to activate host cell phagocytosis (Brittingham et al., 1995), no parasite ligand and host cell receptor pair has been identified so far (Ueno and Wilson, 2012; Rhaiem and Houimel, 2016). Since *Leishmania* MPs can infect most phagocytic cell types we could not screen peptides for a specific host cell population, instead we screened peptides for homogeneous *Leishmania* MPs. As expected, we selected species-specific peptides from each screening. Given the complexity of the library (1.5 × 10^9^), if the selection were not specific, the probability of isolating phages displaying the same peptide would be infinitely small. Thus, while the frequencies are not high at face value, the existence of phages displaying the same peptide isolated multiple times, indicate that such peptide must have affinity for the parasite. Along the same line of thought, the higher the number of phages displaying the same peptide that was selected, the higher the probability that such peptide has a higher affinity to the parasite. We observed that the diversity in phage enrichment may be attributed to differences in the parasite surface. We hypothesize that La1 and Li1 peptides are structural mimics of two different host cell surface molecules or two different epitopes on the same host cell surface molecule. Further studies using these peptides may provide insights into the differences between the two *Leishmania* species and their diverse clinical manifestations. Additionally, they can enhance our understanding of drug target specificity, distinguishing between species-specific and possible universal mechanisms, ultimately advancing our knowledge of *Leishmania* pathogenesis and treatment strategies.

Of note, La1 and Li1 peptides behave differently. Competitive inhibition assays using recombinant phages and synthetic peptides clearly confirm that La1 phage and La1 synthetic peptide selectively inhibit *L. amazonensis* MP-host cell interaction. On the other hand, Li1 phage and Li1 synthetic peptide inhibit MP-host cell interaction for both *L. amazonensis* and *L. infantum*. Although, phage binding assays do not show Li1 phage binding to *L. amazonensis* MP surface and Li1 phage binding to *L. amazonensis* MP surface, synthetic peptide binding assays clearly confirms the results of competitive inhibition assays using recombinant phages and synthetic peptides. Discrepancy of phage and peptide binding assays can be attributed to the size (∼1 μm-long) of the phage particle. The extensive washing step can remove phage particles with moderate strength binding to the MP surface molecule. However, competitive inhibition assays co-incubate candidate phages and MPs in the host cell culture, and washing steps come after the incubation time. Therefore, washing steps do not remove phages on the MP surface, however, can remove weakly bound MPs on the host cell surface. Microscopic observation has shown that peptide binding does not inhibit MP mobility. Moreover, we observed that parasites infectivity was not affected during 8-h incubation of MPs with peptides for *in vitro* inhibition experiments (data not shown), which implies that peptide binding does not affect parasite motility or viability, suggesting that inhibition is related to MP-host cell interaction.

Importantly our *in vivo* experiments confirm the results from *in vitro* inhibition assays: Li1 peptide binding to *L. infantum* MPs inhibits VL development in BALB/c mice, however, no inhibition was observed by La1 peptide. We inoculated MPs and peptide into the mouse peritoneum. Inhibition of 84% parasite growth in the mouse liver and spleen can be attributed to the initial inhibition of MP infection in the mouse peritoneum by Li1 peptide binding. We do not expect that the peptides affect the development of parasites inside the cells, as intracellular parasites have no access to the peptides. Therefore, the use of peptides for post infection treatment cannot be considered. Inhibition of VL development in the spleen was clearer than in the liver because parasite concentration was significantly higher in the spleen than in the liver (compare Li1scr treated groups in the liver and the spleen in Figure 5) since the proportion of phagocytic host cells in the spleen is higher than in the liver. Since Li1 peptide inhibits MP-host cell interaction for both species, it is expected that Li1 peptide mimics a common surface molecule on all phagocytic cells. On the other hand, La1 peptide may mimic a surface molecule of residential skin macrophages. As inhibition of parasite load using 0.2 mg/ml peptide was highly significant, we did not test the 0.5 mg/ml peptide treatment used for *in vitro* experiments.

In summary, we identified the La1 peptide that specifically inhibits L. amazonensis MP infection of host cells and the Li1 peptide that inhibits L. amazonensis and L. infantum MP infection. The Li1 specifically inhibits *L. amazonensis* MP infection into host cells and Li1 peptide that inhibits *L. amazonensis* and *L. infantum* MP infection. The Li1 peptide significantly attenuates development of leishmaniasis in mice. These findings open the way for the development of new strategies to prevent and treat leishmaniasis, an important neglected disease. Identification of the target molecules on the MP surface to which the La1 and Li1 peptides bind will lead to the identification of parasite ligands, which in turn, will serve as promising *Leishmania* vaccine candidates. The La1 and Li1 peptides may be applied for early intervention of MP infection at the sand fly bite site and further peptide application can be tested for amastigote parasite stage for inhibition of disease manifestation. Further, it will be important to assess the effectivity of these peptides to prevent infection by additional species of *Leishmania*.

## Data availability statement

The original contributions presented in the study are included in the article/supplementary material, further inquiries can be directed to the corresponding author.

## Ethics statement

Ethical approval was not required for the studies on humans in accordance with the local legislation and institutional requirements because only commercially available established cell lines were used. The animal study was approved by Institutional Animal Care and Use Committee, Mercer University. The study was conducted in accordance with the local legislation and institutional requirements.

## Author contributions

JV: Data curation, Formal Analysis, Investigation, Methodology, Software, Validation, Visualization, Writing – original draft, Writing – review and editing. MG: Validation, Writing – review and editing. MJ-L: Funding acquisition, Methodology, Resources, Supervision, Writing – review and editing. S-JC: Conceptualization, Data curation, Formal Analysis, Investigation, Methodology, Project administration, Resources, Software, Supervision, Validation, Visualization, Writing – original draft, Writing – review and editing.
